# The Efficacy and Safety of GF101 and Its Antioxidant Effect on In Vitro Fertilization Outcomes: A Double-Blind, Non-Inferiority, Randomized, Controlled Trial with Coenzyme Q10

**DOI:** 10.3390/antiox13030321

**Published:** 2024-03-06

**Authors:** So Yeon Shin, Hye Kyung Yoon, Jee Hyun Kim, Ji Hyang Kim, Chan Park, Dong Hee Choi, Young Dong Yu, Ji Eun Shin, Hwang Kwon

**Affiliations:** 1 Department of Obstetrics and Gynecology, CHA Fertility Center Bundang, 59, Yatap-ro, Bundang-gu, Seongnam-si 13496, Gyeonggi-do, Republic of Korea; syshin88@chamc.co.kr (S.Y.S.); haekyungy@chamc.co.kr (H.K.Y.); jeehyun678@cha.ac.kr (J.H.K.); bin0902@chamc.co.kr (J.H.K.); ifchan424@cha.ac.kr (C.P.); dhchoimc@chamc.co.kr (D.H.C.); 2 Department of Urology, Bundang CHA Hospital, College of Medicine, CHA Medical University, Seongnam-si 13496, Gyeonggi-do, Republic of Korea; a183046@chamc.co.kr

**Keywords:** antioxidant, coenzyme Q10, superoxide dismutase, in vitro fertilization, GF101

## Abstract

(1) Background: Oxidative stress adversely affects fertility by impairing oocyte fertilization potential, primarily due to meiotic segregation errors and cohesion loss. Superoxide dismutase (SOD) and Coenzyme Q10 (CoQ10) are prominent antioxidants known to mitigate oxidative damage. (2) Methods: This study recruited 86 patients undergoing in vitro fertilization (IVF) at a single center for a 12-week, randomized, double-blind, active-comparator-controlled trial. Participants were allocated into two groups: one receiving CoQ10 as an antioxidant (the CoQ10 group) and the other receiving GF *Bacillus* antioxidative enzyme SOD (the GF101 group). The primary endpoints were changes in serum oxidative markers (SOD and catalase) and IVF outcomes, including clinical pregnancy, miscarriage, and live birth rates. Follicular fluid (FF) SOD and catalase concentrations on the day of retrieval, the metaphase II (MII) oocyte rate, the fertilization rate, and lipid profiles were measured. (3) Results: Initially, 86 patients were enrolled, with 65 completing the protocol (30 in the GF101 group and 34 in the CoQ10 group). There were no significant differences between the GF101 and CoQ10 groups in serum SOD (*p* = 0.626) and catalase levels (*p* = 0.061) over 12 weeks. However, within the GF101 group, a significant increase in serum catalase from baseline to 12 weeks was observed (*p* = 0.004). The non-inferiority analysis for IVF outcomes indicated risk differences in the clinical pregnancy rate, live birth rate, and miscarriage rate of −6.27% (95% CI: −30.77% to 18.22%), −1.18% (95% CI: −25.28% to 22.93%), and −13.49% (95% CI: −41.14% to 14.15%), respectively, demonstrating non-inferiority for the GF101 group. Furthermore, the GF101 group experienced significant reductions in total cholesterol (*p* = 0.006) and low-density lipoprotein (LDL) levels (*p* = 0.009) in intra-group comparisons, with both groups exhibiting comparable safe profiles. (4) Conclusions: GF101 may be non-inferior to CoQ10 in treating infertility in women and potentially offers additional benefits for women with dyslipidemia.

## 1. Introduction

Oxidative stress refers to a state of an imbalance between the production of reactive oxygen species (ROS) and antioxidant defenses in aerobic organisms [1]. Oxidative stress also affects fertility, and ROS accumulation can cause oocyte damage through several pathways. ROS accumulation can adversely affect the fertilization potential of oocytes by reducing membrane fluidity through the lipid peroxidation of the plasma membrane [2,3]. In addition, oxidative damage contributes to meiotic segregation errors and cohesion loss [4], particularly with increasing maternal age, leading to diminished fertilization rates, compromised embryo quality, and potential abnormalities in offspring [5].

Superoxide dismutases (SODs) are a group of metalloenzymes that directly decompose the superoxide anion, a major oxidative radical [6]. SOD acts as an antioxidant by catalyzing the dismutation of superoxide into hydrogen peroxide and oxygen, and it exists in three isoforms in humans: cytosolic (defined as SOD1), mitochondrial (defined as SOD2), and extracellular SOD (defined as SOD3) [7,8,9]. The three isoforms, respectively, present different structures: both SOD1 and SOD3 have catalytic centers with Copper (Cu) and Zinc (Zn), but SOD1 is localized to intracellular cytoplasmic compartments, and SOD3 is localized to extracellular elements [7]. SOD2 has Manganese (Mn) as a cofactor and is localized in the mitochondria of aerobic cells [10,11]. Our target drug, GF101, also uses Mn as a cofactor, corresponding to SOD2 [7]. The intracellular isoforms of SOD hardly bind to the endothelium and are relatively short-lived, so extracellular SOD has recently been considered as a candidate for therapeutic agents [7]. SOD needs optimal pharmacokinetics because orally administered SOD has low bioavailability due to its high molecular weight and low intestinal permeability [12]. Therefore, SOD mimetics, synthetic antioxidant enzymes, were developed with the pharmacological characteristics of low molecular weight, better intestinal permeability, longer circulating half-life, and lower antigenicity [12]. In previous studies, SOD exerted a protective effect on sperm viability and improved the development of embryos [13]. When SOD was applied to culture media, it improved sperm motility, reduced oocyte apoptosis, and increased the number and quality of embryos [11,13,14].

GF101, an antioxidant enzyme SOD derived from the *Bacillus amyloliquefaciens* (*B. amyloliquefaciens*) GF423 strain (KCTC 13222BP) (BiomLogics Inc., Seoul, Republic of Korea), has demonstrated therapeutic efficacy in various diseases, such as inflammatory bowel diseases, diabetes, and atherosclerosis [15,16,17,18,19]. This is achieved through the inhibition of inflammatory mechanisms and the removal of ROS compounds, facilitated by oral administration [12,15,20,21]. Unlike conventional antioxidants that act through secondary mechanisms, GF101 converts two superoxide anions into one molecule of hydrogen peroxide and one molecule of O2 [22].

Antioxidant supplements, like Coenzyme Q10 (CoQ10), are often used by patients with subfertility to mitigate oocyte damage and enhance the quality of oocytes. CoQ10, a molecule found in the hydrophobic domain of all cell membranes, functions as an electron and proton carrier in the mitochondrial respiratory chain and participates in ATP synthesis [1,23]. It also acts as an antioxidant, protecting cell membranes from lipid peroxidation and boosting the activity of antioxidant enzymes [1,23]. Oocyte aging is accompanied by mitochondrial dysfunction, associated with decreased oxidative phosphorylation and reduced adenosine tri-phosphate (ATP) levels [23]. The impaired mitochondrial performance created by suboptimal CoQ10 availability can drive age-associated oocyte deficits, causing infertility [23]. CoQ10 is thought to be able to delay oocyte aging and prevent the decline in reproductive function [24]. Additionally, CoQ10 increases the number of cumulus cells surrounding the oocyte, reducing oocyte apoptosis and increasing developmental competence, ultimately improving reproductive performance [23].

We hypothesized that GF101 could enhance antioxidant capabilities and improve pregnancy outcomes in patients undergoing in vitro fertilization (IVF). Thus, we conducted a double-blind, non-inferiority, randomized, controlled trial with an active comparator, CoQ10. The purpose of this study is to compare the efficacy and safety of these two antioxidants with different mechanisms of action in patients with subfertility.

## 2. Materials and Methods

### 2.1. Trial Design and Participants

This investigator-initiated trial was a single-center, double-blind, randomized, active-controlled trial from 28 April 2021 to 23 August 2022 to evaluate the efficacy of GF101 on antioxidant capacity and its influence on in vitro fertilization outcomes in women with subfertility. The concept and procedures of this study were approved by our Institutional Review Board (IRB number: 2020-11-016), and all patients provided written informed consent. The key eligibility criteria included (1) age from 20 years to 49 years, (2) body mass index (BMI) ranging from 18 kg/m^2^ to 30 kg/m^2^, (3) women who plan to undergo IVF procedures. The exclusion criteria were as follows: (1) those with an uncontrolled endocrine disease or medical disease; (2) those who had smoked excessively within 3 months as of visit 1: smoked 10 cigarettes a day or, in the case of electronic cigarettes, inspired about 100 times a day; (3) those who had consumed excessive alcohol within 3 months as of visit 1 (340 g/week, about 7 bottles of soju a week, 1 bottle a day) or were alcoholic; (4) women with moderate gynecological disease (endometriosis, deformation of the uterine lumen due to submucosal uterine fibroids, polyps, pelvic inflammatory disease, uterine malformation, and hydrosalpinx), although participation was possible if uterine fibroids and polyps were not related to infertility; (5) men with seminal duct obstruction; (6) men who needed testicular sperm extraction/testicular sperm aspiration; and (7) those who were receiving other auxiliary treatment, Oriental medicine treatment, etc., for the purpose of infertility treatment. Detailed information on inclusion and exclusion criteria is described in Supplementary Table S1.

### 2.2. Experimental Procedures

Patients were randomly assigned in a 1:1 ratio to receive either GF101 or Coenzyme Q10, both at doses of 500 IU twice daily for 12 weeks [25]. The study treatment was discontinued in cases deemed at risk by the investigator, unacceptable adverse events, or the patient’s refusal to continue the study treatment. The discontinuation of the SOD supplement was considered by the investigator if the patient was likely to cancel their IVF cycle or no oocyte was retrieved. Patients were thus allocated to two groups: the GF101 group (patients who received GF101) and the CoQ10 group (patients who received CoQ10). During five hospital visits (on randomization day and at 2, 4, 8, and 12 weeks), we collected baseline characteristics, serum oxidative stress markers, lipid profiles, inflammation markers, information regarding the number of retrieved oocytes, IVF outcomes, and adverse events.

For the IVF procedure, ovarian stimulation was performed by two different physicians considering individual characteristics, including the patient’s age, ovarian reserve, and BMI, and follicle growth was monitored by ultrasound examination at 3–4 days intervals. Oocyte retrieval was performed 35–37 h after ovulation was triggered by human chorionic gonadotropin administration. On oocyte retrieval day, the dominant follicle/s was/were aspirated into an empty tube up to a total volume exceeding 5 mL following oocyte isolation. The FF was centrifuged at 1500× *g* for 7 min and was frozen and stored at –80 °C until analysis. The follicular fluid was collected. Embryos were created by fertilizing oocytes and sperms through conventional IVF or ICSI. Embryos were transferred into the uterus under transabdominal ultrasound guidance.

### 2.3. Outcome Measures

Since the purpose of GF101 administration in this study was to improve pregnancy outcomes by increasing serum SOD activity, the primary endpoints were changes in serum oxidative stress markers (SOD and catalase) over 12 weeks and IVF outcomes, including the clinical pregnancy rate, live birth rate, and miscarriage rate. 

Secondary endpoints included changes in follicular fluid (FF) SOD and catalase concentrations at 2 weeks, lipid profile alterations over 12 weeks (total cholesterol, high-density lipoprotein (HDL), low-density lipoprotein (LDL), and triglycerides), changes in inflammation markers (tumor necrosis factor (TNF)-α, interleukin (IL)-1β, IL-6, high-sensitive C reactive protein (hs-CRP), natural killer (NK) cell activities), and clinical IVF outcomes (MII per total oocyte rate, fertilization rate, good-quality embryo rate, total pregnancy rate, implantation rate). SOD is a metalloprotein that scavenges O_2_^●−^ and converts it into hydrogen peroxide (H_2_O_2_) and molecular oxygen (O_2_) [26]. Subsequently, H_2_O_2_ is reduced to water by catalase [26]. Thus, serum levels of SOD and catalase were included as antioxidative markers in our study. In a previous study by J. Hwang et al., it was demonstrated that *B. amyloliquefaciens* SOD inhibited inflammation and apoptosis through the suppression of the p38-MAPK/NF-kB signaling pathway [27]. Based on these findings, we incorporated several inflammatory markers into our analysis. Furthermore, antioxidants have been shown to improve lipid profiles in the existing literature. In light of this evidence, we also investigated lipid profiles in the current study to assess the potential ameliorative effects of antioxidants on lipid metabolism [28,29,30].

Safety assessment involved collecting adverse events (AEs), serious AEs, causal relationships, clinical laboratory measurements, and vital signs from enrollment to discontinuation. Meanwhile, we investigated baseline characteristics, including age, BMI, infertility factor (male cause, bilateral tubal obstruction, decreased ovarian response, unexplained, others), alcohol history (alcohol-drinking history within 3 months prior to visit), smoking history (smoking history within 3 months prior to visit), past medical history within 1 month prior to visit (including cardiac disorders; congenital, familial, and genetic disorders; endocrine disorders; gastrointestinal disorders; general disorders and administration site conditions; infections and infestations; abnormal laboratory findings; metabolic and nutritional disorders; neoplasm [such as uterine leiomyoma]; renal and urinary disorders; reproductive system and breast disorders; respiratory, thoracic, and mediastinal disorders; vascular disorders [such as hypertension]; and any surgical history), and medication history within the past 1 month.

### 2.4. Statistical Analysis

Statistical analysis and visualization were conducted using Python (version 3.11.5., Python Software Foundation, Wilmington, DE, USA) with matplotlib (version 3.7.2.). The continuous variables were expressed to two decimal places. A normal distribution was confirmed using the Kolmogorov–Smirnov test. To assess the adequacy of the sample size, we re-confirmed it using post hoc power analysis (with effect size 0.8) to justify the adequate sample size after the drop-out process. Data homogeneity or heteroscedasticity was determined by Student’s *t*-test (for paired or independent means) for continuous variables and the chi-square or Fisher’s exact test for categorical variables, as appropriate. For non-inferiority assessment, a non-inferiority margin was defined as −1.0. Statistical significance was considered at two-tailed *p*-values < 0.05.

## 3. Results

### 3.1. Baseline Demographic Characteristics

**Figure 1:**
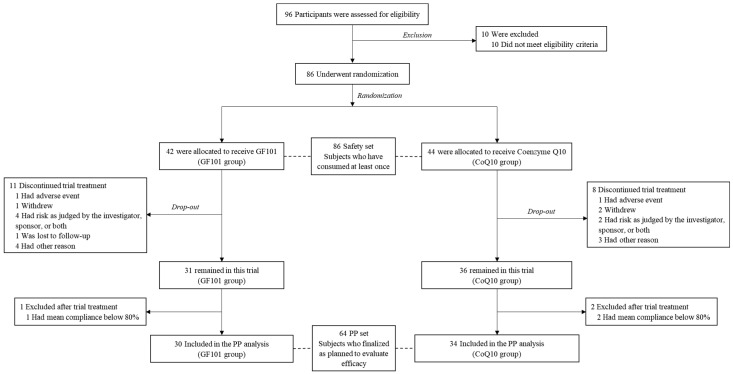
Flow chart of study design.

A total of 86 participants were randomly assigned to the GF101 group (n = 42) and the CoQ10 group (n = 44) after the assessment of eligibility criteria. These participants (n = 86) were included as a safety set, having consumed GF101 or CoQ10 at least once (post hoc power = 0.95). The treatment was discontinued for 19 participants (n = 11 in the GF101 group and n = 8 in the CoQ10 group), and a further 3 were excluded due to compliance below 80% (one in the GF101 group and two in the CoQ10 group) (Supplementary Table S2). We finally defined the per-protocol (PP) set as the GF101 group (n = 30) and the CoQ10 group (n = 34) (post hoc power = 0.88) (Figure 1). 

The mean age was 36.30 years in the GF101 group and 35.38 years in the CoQ10 group, respectively, with no significant differences (*p* = 0.345). There was no statistical significance for the infertility factor (*p* = 0.190), alcohol history (*p* = 0.679), smoking history (*p* = 1.00), past medical history (*p* = 0.542), and medication history (*p* = 0.736) between the two groups. Detailed baseline characteristics are described in Table 1. 

### 3.2. Serum Oxidative Stress Markers and IVF Outcomes

**Figure 2:**
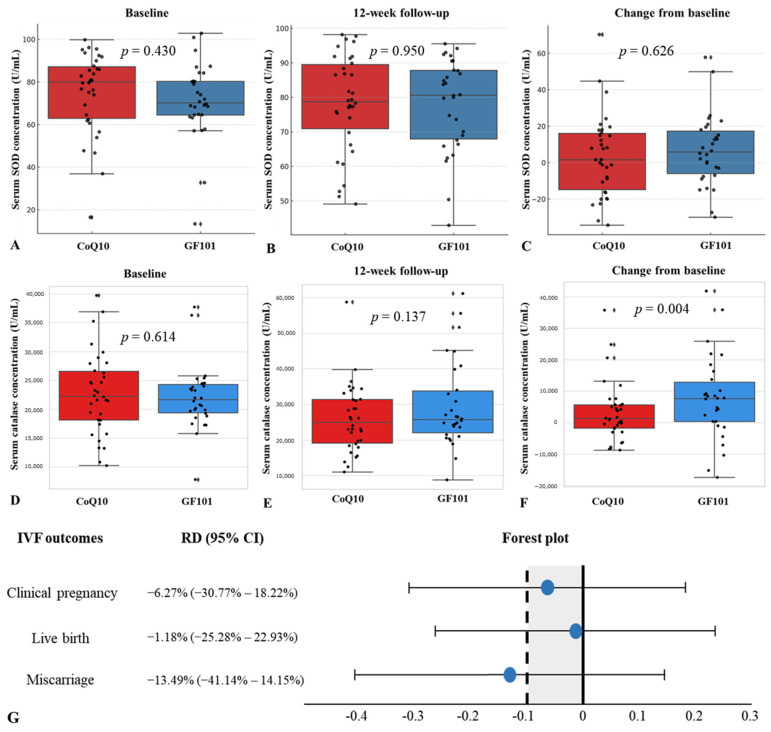
Comparison of serum SOD concentration between the CoQ10 group and the GF101 group at baseline (**A**), at 12 weeks (**B**), and change from baseline (**C**). Comparison of serum catalase concentration between the CoQ10 group and the GF101 group at baseline (**D**), at 12 weeks (**E**), and change from baseline (**F**). Non-inferiority analysis of clinical pregnancy rate, live birth rate, and miscarriage rate between the CoQ10 group and the GF101 group (**G**).

We defined primary outcomes as changes in serum oxidative markers, SOD and catalase, and IVF outcomes, including the clinical pregnancy rate, live birth rate, and miscarriage rate. The mean baseline serum SOD concentration was 71.22 (U/mL) in the GF101 group and 74.89 (U/mL) in the CoQ10 group (*p* = 0.430) (Figure 2A). The mean 12-week follow-up serum SOD concentration was 77.91 (U/mL) in the GF101 group and 77.71 (U/mL) in the CoQ10 group (*p* = 0.950) (Figure 2B). The change in serum SOD concentration over 12 weeks was 6.69 (U/mL) in the GF101 group and 2.81 (U/mL) in the CoQ10 group, with no statistical significance (*p* = 0.626) (Figure 2C). The average serum catalase concentration was 22,009.73 (U/mL) in the GF101 group and 22,709.22 in the CoQ10 group (*p* = 0.614) (Figure 2D), and after 12 weeks, the serum catalase concentration showed no statistical differences between the two groups (*p* = 0.137) (Figure 2E). However, the GF101 group showed a significant increase in serum catalase from baseline to 12 weeks (*p* = 0.004) (Figure 2F).

For the non-inferiority analysis of IVF outcomes, the risk differences of the clinical pregnancy rate, live birth rate, and miscarriage rate were −6.27% (95% CI: −30.77% to 18.22%), −1.18% (95% CI: −25.28% to 22.93%), and −13.49% (95% CI: −41.14% to 14.15%), respectively (Figure 2G).

### 3.3. FF Oxidative Stress Markers, Oocyte Maturation, and Lipid Profiles

FF SOD (*p* = 0.519) and catalase (*p* = 0.490) showed no significant differences between the two groups at 2 weeks, collected on the day of oocyte retrieval. Lipid profiles were observed as not significantly different between the two groups at baseline and the 12-week follow-up (all *p*-values > 0.05). However, the GF101 group exhibited significant reductions in total cholesterol (204.80 to 189.87, *p* = 0.006) and LDL (128.30 to 114.20, *p* = 0.006) from baseline to after 12 weeks, unlike the CoQ10 group. Inflammation markers and NK cell activity showed no significant differences between the two groups (all *p*-values > 0.05), as shown in Table 2.

Oocyte retrieval was conducted at 2 weeks. We evaluated the cycle outcomes of the GF101 and CoQ10 groups. The mean number of retrieved oocytes was 8.07 in the GF101 group and 7.94 in the CoQ10 group, with no significance (*p* = 0.933). The mean number of MII oocytes was 7.90 in the GF101 group and 7.79 in the CoQ10 group, which are not significantly different (*p* = 0.854). In addition, there were no statistical differences in MII/total retrieved oocytes, the fertilization rate, the good-quality embryo rate, the total pregnancy rate, the implantation rate, the clinical pregnancy rate, the live birth rate, and the miscarriage rate (all *p*-values > 0.05) (Table 3).

### 3.4. Safety Analysis

We describe detailed AEs for this trial in Table 4. In the safety set, adverse events (AEs) were similarly reported in both groups (54.76% in the GF101 group and 54.55% in the CoQ10 group, *p* = 0.984). A total of 48 AEs occurred in the GF101 group, and the main adverse reactions were associated with reproductive system and breast disorders (30.95%). In the CoQ10 group, a total of 34 AEs occurred, and the main adverse reactions were also associated with reproductive system and breast disorders (34.09%). The mild, moderate, and severe AE numbers per patient were 1.84, 1.57, and 1.00 in the GF101 and 1.20, 1.14, and 0 in the CoQ10 group, respectively. Only one patient in the GF101 group (2.38%) experienced a serious AE, uterine leiomyoma, which was surgically treated and was shown to be “definitely not related” for its causal relationship. AEs that cannot be ruled out in relation to GF101 or CoQ10 were mostly associated with gastrointestinal disorders.

## 4. Discussion

The decrease in reproductive potential in women experiencing subfertility with advanced maternal age is attributed to less efficient antioxidant defense systems as women age. The accumulation of oxidative stress and the deterioration of oocytes has been well documented [31,32,33,34]. Oocyte mitochondria are the primary source of ROS production, and mitochondrial damage can negatively impact meiotic spindle assembly, which is responsible for chromosomal segregation and oocyte competence. For patients with advanced maternal age undergoing IVF, the consumption of antioxidant supplements is considered to improve IVF outcomes due to the absence of a definitive treatment. 

CoQ10 acts as an effective antioxidant by directly scavenging free radicals and preventing lipid peroxidation and DNA oxidation [35]. A Cochrane systematic review and several meta-analyses have shown that antioxidants improve clinical pregnancy rates and live birth rates compared with placebo or no treatment (OR 1.65 and 1.81, respectively), although the quality of the evidence was low to very low [36,37,38]. In particular, CoQ10 was associated with an increased clinical pregnancy rate with OR 2.49 compared to placebo or no treatment [38]. In contrast, evidence of the potential effect of SOD on antioxidative mechanisms and reproductive outcomes was scarce, despite the shared antioxidative mechanisms between SOD and CoQ10. In this study, we performed a single-center, double-blind, randomized, active-controlled trial to evaluate the efficacy, IVF outcomes, and safety of GF101 compared to CoQ10. 

In this study, we measured oxidative stress markers, SOD and catalase, in both the serum and FF to determine the direct improvement of the antioxidative reaction in the cumulus–oocyte complex. Although SOD and catalase did not show a significant difference between the two groups, the IVF outcomes, including the clinical pregnancy rate, miscarriage rate, and live birth rate, did not show statistical significance. However, in the intra-group analysis, the GF101 group showed significantly increased serum catalase at 12 weeks from baseline. 

Among the secondary outcomes, although there was no significant difference in the lipid profiles between the two groups, the GF101 group showed significantly reduced total cholesterol and LDL concentration in the intra-group analysis, suggesting that GF101 might be more effective in improving lipid profiles. This may lead to the conclusion that, in IVF patients with dyslipidemia, it may be advantageous to apply GF101 preferentially over CoQ10 without concern for a decrease in IVF outcomes or adverse effects. From the results, we demonstrated that GF101 may not be inferior to CoQ10 in efficacy and safety as an antioxidative supplement.

SOD is an aerobic enzyme that reduces oxidative stress arising from ROS and regulates cellular lifespan and fibrosis [39]. SOD activity is known to decrease with increasing female age [40]. In a previous animal study, SOD knockdown Drosophila showed significantly increased nondisjunction in meiotic prophase compared to the control group due to oxidative damage-induced cohesion loss [4]. Moreover, in the Gal4/UAS system, the induced overexpression of SOD1 and SOD2 in Drosophila oocytes during prophase significantly reduced nondisjunction [41]. Therefore, GF101 could improve oocyte competence and pregnancy outcomes. 

For the mechanisms of antioxidants in human diseases, CoQ10 acts as an antioxidant by removing free radicals, protecting cell membranes from lipid peroxidation, and enhancing the activity of antioxidant enzymes [1,23,42]. On the other hand, SOD directly decomposes the superoxide anion [12]. The superoxide radical, one of the most critical oxidative radicals, is biologically important because it can generate other more reactive species, such as the hydroxyl radical [42,43]. Previous studies have been published on the effects of SOD administration on metabolic disorders such as lipids, and some results support ours that SOD administration improves lipid profiles [43]. This is believed to be caused by SOD preventing a metabolic deficit in skeletal muscles, which is due to hypermetabolism from the production of superoxide during ATP synthesis from glucose and free fatty acids [12,43]. GF101, originating from the mitochondrial SOD (SOD2) isoform, also contributed to ATP, which may potentially improve lipid profiles.

As far as we know, no studies have been conducted to date on the antioxidative effects of any SOD, including GF101, in improving IVF outcomes or fertility as an oral supplement. Other than SOD and CoQ10, supplementary oral antioxidants that have been studied for their effectiveness in improving fertility outcomes include myo-inositol, L-arginine, vitamins, melatonin, and omega-3 [38]. The mechanisms through which supplementary antioxidants enhance fertility are diverse. Myo-inositol improves ovarian reserve, and L-arginine enhances endometrial blood flow [38]. Vitamin E helps with epithelial growth in blood vessels and in the endometrium, and vitamin D increases pregnancy rates, especially in women with polycystic ovarian syndrome (PCOS), by lowering hyperandrogenism [44]. In the Cochrane review, melatonin was found to increase the clinical pregnancy rate like CoQ10, but no difference was observed based on the dosage [38]. Therefore, our research also provides a future subject for SOD’s influence on hormonal diseases in women.

As far as we know, this is the first study to analyze IVF outcomes using *Bacillus* SOD, GF101, as an oral supplement. However, this study has several limitations. Firstly, we performed a non-inferiority test compared to an active comparator, CoQ10, instead of recruiting control groups, which could make it difficult to ascertain the direct efficacy of GF101. This is because most patients undergoing IVF did not want to waste their limited reproductive lifespan in a sub-optimal condition and had a strong desire for proactive add-ons during IVF treatment. In addition, this study did not include an intention-to-treat analysis but solely conducted a PP analysis, potentially causing statistical limitations. Finally, this study was conducted on a patient cohort recruited solely from a single IVF center, and the sample size of the study was small. Furthermore, the wide range of patients may have impacted the laboratory findings, oocyte quality, and pregnancy rate because age is an important factor for the prognosis of IVF outcomes. Although a wide age range was observed between the two groups, there was no statistical difference in age between the two groups, which can be compared for our results. We included patients with a social history (alcohol history and smoking history) and only excluded excess smokers (≥10 cigarettes/day; for electronic cigarettes, approximately 100 inhalations/day) and alcohol consumers (≥340 g/week, equivalent to about 7 bottles of soju per week or 1 bottle per day, or alcoholism) due to previous approval up to these levels in patients. Considering that the antioxidant effects were influenced by alcohol and smoking, the influence of alcohol and smoking needs to be addressed as a new topic. Therefore, the results of this study need to be strengthened through future multi-center, large-scale research.

## 5. Conclusions

Our randomized controlled trial has demonstrated that GF101 possesses efficacy comparable to that of CoQ10 in treating women with infertility. Furthermore, GF101 was observed to significantly reduce total cholesterol and LDL levels, indicating its potential enhanced benefit for women with dyslipidemia. The IVF outcomes following GF101 administration may not be inferior to those observed with CoQ10. Therefore, GF101 may be considered an alternative option as an antioxidant agent. 

## Figures and Tables

**Table 1:** Baseline characteristics in this study.

Variables	GF101 (n = 30)	CoQ10 (n = 34)	*p*-Value
Age, mean ± S.D. (years)	36.30 ± 4.25	35.38 ± 3.46	0.345
Age, range (years)	29–49	29–42	
BMI, mean ± S.D. (kg/m^2^)	24.57 ± 3.37	22.79 ± 3.52	0.044
BMI, range (kg/m^2^)	17.20–29.90	19.00–29.80	
Infertility factor, n (%)			0.19
Male	3 (10.71%)	6 (19.35%)	
BTO	4 (14.29%)	3 (9.68%)	
DOR	0 (0%)	4 (12.90%)	
Unexplained	6 (21.43%)	8 (25.81%)	
Others	15 (53.57%)	10 (32.26%)	
Alcohol history, n (%)			0.679
Yes	13 (43.33%)	13 (38.24%)	
No	17 (56.67%)	21 (61.76%)	
Smoking history, n (%)			1.000
Yes	4 (13.33%)	4 (11.76%)	
No	26 (86.67%)	30 (88.24%)	
Past medical history, n (%)			0.542
Yes	24 (80.00%)	25 (73.53%)	
No	6 (20.00%)	9 (26.47%)	
Medication history, n (%)			0.736
Yes	20 (66.67%)	24 (70.59%)	
No	10 (33.33%)	10 (29.41%)	

All the data shown are mean ± standard deviation, range (minimum–maximum), and number (%). The *p*-values are from comparisons between the GF101 group and the CoQ10 group. n, number; S.D., standard deviation; BTO, bilateral tubal occlusion; DOR, diminished ovarian reserve.

**Table 2:** Secondary outcome measures for efficacy of GF101 compared to CoQ10.

Variables	GF101 Group	CoQ10 Group	*p*-Value
Oxidative stress markers in FF			
FF SOD at 2 weeks, mean ± S.D. (U/mL)	1.03 ± 2.02	0.54 ± 0.12	0.519
FF catalase at 2 weeks, mean ± S.D. (U/mL)	9.22 ± 7.05	10.68 ± 8.42	0.490
Lipid profiles			
Total cholesterol, mean ± S.D. (mg/dL)			
Baseline	204.80 ± 40.46	192.15 ± 34.80	0.184
12-week follow-up	189.87 ± 27.82	182.88 ± 34.54	0.381
Change from baseline	−14.93 ± 27.51	−9.26 ± 38.48	0.323
*p*-value for paired	0.006	0.170	
HDL, mean ± S.D. (mg/dL)			
Baseline	57.82 ± 11.09	64.40 ± 11.95	0.028
12-week follow-up	55.72 ± 12.20	63.70 ± 12.95	0.014
Change from baseline	−2.10 ± 8.96	−0.39 ± 9.37	0.464
*p*-value for paired	0.210	0.811	
LDL, mean ± S.D. (mg/dL)			
Baseline	128.30 ± 35.20	115.03 ± 31.86	0.119
12-week follow-up	114.20 ± 32.19	107.74 ± 30.14	0.492
Change from baseline	−14.10 ± 27.50	−7.29 ± 33.95	0.336
*p*-value for paired	0.009	0.219	
TG, mean ± S.D. (mg/dL)			
Baseline	118.40 ± 77.79	111.85 ± 116.53	0.253
12-week follow-up	145.03 ± 106.51	96.41 ± 49.94	0.046
Change from baseline	26.63 ± 90.51	−14.39 ± 122.50	0.573
*p*-value for paired	0.118	0.505	
Inflammation markers			
TNF-α, mean ± S.D. (pg/mL)			
Baseline	0.55 ± 0.22	0.64 ± 0.41	0.443
12-week follow-up	0.53 ± 0.19	0.54 ± 0.27	0.821
Change from baseline	−0.02 ± 0.14	−0.10 ± 0.33	0.672
*p*-value for paired	0.387	0.093	
IL-1β, mean ± S.D. (U/mL)			
Baseline	0.08 ± 0.04	0.08 ± 0.05	0.576
12-week follow-up	0.08 ± 0.04	0.08 ± 0.06	0.626
Change from baseline	0.00 ± 0.04	0.01 ± 0.07	0.778
*p*-value for paired	0.668	0.549	
IL-6, mean ± S.D. (U/mL)			
Baseline	1.75 ± 1.20	1.65 ± 0.93	0.989
12-week follow-up	2.30 ± 1.57	1.79 ± 0.98	0.12
Change from baseline	0.55 ± 1.63	0.14 ± 1.00	0.439
*p*-value for paired	0.074	0.427	
hs-CRP, mean ± S.D. (U/mL)			
Baseline	0.19 ± 0.18	0.13 ± 0.13	0.107
12-week follow-up	0.38 ± 0.54	0.14 ± 0.13	0.021
Change from baseline	0.19 ± 0.55	0.01 ± 0.15	0.636
*p*-value for paired	0.067	0.576	
Immune cell activity			
NK cell activity (U/mL)			
Baseline	2588.77 ± 2968.26	2378.86 ± 3392.39	0.687
12-week follow-up	1899.59 ± 3344.97	1784.31 ± 6589.66	0.931
Change from baseline	−689.17 ± 4095.96	−594.54 ± 7686.04	0.431
*p*-value for paired	0.364	0.655	

n, number; S.D., standard deviation; FF, follicular fluid; HDL, high-density lipoprotein; LDL, low-density lipoprotein; TG, triglyceride; TNF, tumor necrosis factor; IL, interleukin; hs-CRP, high-sensitive C reactive protein; NK, natural killer.

**Table 3:** In vitro fertilization (IVF) outcome measures of the two groups.

Variables	GF101 Group	CoQ10 Group	*p*-Value
Oocytes at 2 weeks			
Number of retrieved oocytes, mean ± S.D. (n)	8.07 ± 4.23	7.94 ± 3.82	0.933
Number of MII retrieved oocytes, mean ± S.D. (n)	7.90 ± 4.26	7.79 ± 3.75	0.854
Clinical outcomes of IVF			
MII/total retrieved oocyte rate (%)	60.11% (226/376)	54.30% (246/453)	0.093
Fertilization rate (%)	76.36% (239/313)	71.86% (263/366)	0.183
Good-quality embryo rate (%)	81.71% (67/82)	89.11% (90/101)	0.154
Total pregnancy rate (%)	60.71% (17/28)	67.74% (21/31)	0.598
Implantation rate (%)	38.10% (16/42)	51.28% (20/39)	0.268
Clinical pregnancy rate (%)	46.67% (14/30)	52.94% (18/34)	0.803
Live birth rate (%)	40.00% (12/30)	41.18% (14/34)	0.924
Miscarriage rate (%)	14.29% (2/14)	27.78% (5/18)	0.426

MII, metaphase II oocyte; the data shown are % (number). The *p*-values are from comparisons between the GF101 group and the CoQ10 group.

**Table 4:** Safety of GF101 compared to CoQ10 for women with infertility.

Variables	GF101 Group (n = 42)	CoQ10 Group (n = 44)	*p*-Value
AEs			
Total number	48	34	
Experienced patients’ number	23 (54.76%)	24 (54.55%)	0.984
AEs number per patient			
Mild	1.84 (35/19)	1.20 (26/20)	
Moderate	1.57 (11/7)	1.14 (8/7)	
Severe	1.00 (2/2)	0 (0/0)	
Serious AEs			
Experienced patients’ number	1 (2.38%)	0 (0%)	0.488
Causal relationship, number (events/patients)			
Definite related	0/0	0/0	
Probably related	0/0	1/1	
Possibly related	1/1	0/0	
Probably not related	1/1	0/0	
Definitely not related	40/22	33/23	
Unknown	6/1	0/0	
AEs that cannot be ruled out in relation to target drugs, number (events/patients)	7/2	1/1	
Gastrointestinal disorders	3/2	1/1	
Abdominal pain	1/1	0/0	
Upper abdominal pain	0/0	1/1	
Diarrhea	1/1	0/0	
Nausea	1/1	0/0	
Abnormal laboratory findings	2/1	0/0	
ALT increased	1/1	0/0	
Blood cholesterol increased	1/1	0/0	
Metabolism and nutritional disorders	2/1	0/0	
Hypertriglyceridemia	2/1	0/0	

AE, adverse event.

## Data Availability

The data presented in this study are available on request.

## Supplementary Materials

The following supporting information can be downloaded at https://www.mdpi.com/article/10.3390/antiox13030321/s1: Table S1: Detailed information on the inclusion and exclusion criteria of the study population. Table S2: Analysis set and detailed description of participants in the study who were excluded due to discontinuation or non-compliance.
