# Supplementary material for: The Efficacy and Safety of GF101 and Its Antioxidant Effect on In Vitro Fertilization Outcomes: A Double-Blind, Non-Inferiority, Randomized, Controlled Trial with Coenzyme Q10
Source: Antioxidants (Basel). 2024 Mar 6;13(3):321. doi: 10.3390/antiox13030321 (PMC10967835; doi:10.3390/antiox13030321)
Supplement: Supplementary file 1 [file antioxidants-13-00321-s001.zip › antioxidants-2864544-supplementary.pdf]

Supplementary Table S1. Detailed information for the inclusion and exclusion criteria of study population

Methods	Description
Inclusion criteria	The inclusion criteria were as follows: (1) age between 20 and 50 years and (2) body mass index (BMI) greater than 18kg/m^2 and less than 30kg/m^2, (3) female planned ovarian stimulation for IVF, (4) Individuals who signed an Informed Consent form.
Exclusion criteria	Individuals with any of the following conditions were excluded: (1) Individuals with uncontrolled endocrine or internal medical condition, (2) Excessive smokers within 3 months prior to visit 1 (≥10 cigarettes/day; for electronic cigarettes, approximately 100 inhalations/day) (3)excessive alcohol consumers within 3 months prior to visit (≥340 g/week, equivalent to about 7 bottles of soju per week or 1 bottle per day), or those suffering from alcoholism. (4) women with moderate gynecological condition (endometriosis, submucosal uterine fibroids causing deformation of the uterine cavity, polyps, pelvic inflammatory disease, uterine malformations, hydrosalpinx), except in cases where the investigator deemed uterine fibroids and polyps unrelated to infertility. (6) Male partner requiring TESE/TESA procedures (7) Individuals receiving alternative treatments for infertility, and (8) Others deemed unsuitable by the investigator for various reasons.

BMI, body mass index; IVF, in-vitro fertilization; TESE, testicular sperm extraction; TESA, testicular sperm aspiration.

Supplementary Table S2. Analysis set and detailed description of discontinuation and non-compliance participants in the study

Variables	GF101<br>N (%)	CoQ10<br>N(%)	Total<br>N(%)
**Safety set**	42 (100.0)	44(100.0)	86(100.0)
**Full Analysis set**	31 (73.8)	36 (81.8)	67(77.9)
**Drop out**			
-IVF cycle cancellation	1(2.38)	1(2.27)	2(2.33)
-consent withdrawal	1(2.38)	2(4.55)	3(3.49)
-no oocyte retrieved	3(7.14)	2(4.55)	5(5.81)
-lost to follow up	1(2.38)	0(0.00)	1(1.16)
-risk judged by investigator	4(9.52)	2(4.55)	6(6.98)
-adverse event	1(2.38)	1(2.27)	2(2.33)
**Per Protocol set**	30 (96.8)	34(94.4)	64(95.5)
-mean compliance below 80%	1(3.23)	2(5.56)	3(4.48)

N, number
